# PS1 regulates processing and angiogenic signalling of VEGFR2; effects of FAD mutants

**DOI:** 10.1002/alz.092786

**Published:** 2025-01-03

**Authors:** Poulomi Dey, Rukmani Pandey, Amira Zarrouk, Glikeria Tzikas, Anastasios Georgakopoulos, Nikolaos K. R K Robakis

**Affiliations:** ^1^ Icahn School of Medicine at Mount Sinai Medical Center, New York, NY USA

## Abstract

**Background:**

Presenilin1 (PS1)/γ‐secretase cleaves within the transmembrane domain of numerous receptor substrates. Mutations in PS1 have implications on the catalytic subunit of γ‐secretase decreasing its activity and becoming a potential causative factor for Familial Alzheimer’s Disease (FAD). This work studies the role of PS1/γ‐secretase on the processing, angiogenic signaling, and functions of VEGFR2 and the effects of PS1 FAD mutants on the γ‐secretase‐mediated epsilon cleavage of VEGFR2.

**Method:**

HEK293T cells expressing WT‐PS1 or PS1‐FAD mutants (M146V, I213T, E280A, G384A, L166P, E120K, A246E) were prepared using a lentivirus‐based transduction system and used in luminescence‐based experiments. PS1‐VEGFR2 co‐immunoprecipitation (Co‐IP) experiments were analyzed on WBs. Bimolecular fluorescence complementation (BiFc) assays were carried out in HEK293T cells overexpressing PS1 or VEGFR2 tagged with half complementary portions of the mVenus and visualized by fluorescence microscopy. The ClusPro web server was used for protein‐protein docking analyses. Anti‐PS1 siRNA technology was applied to downregulate PS1 in endothelial bEnd.3 cells.

**Result:**

Luciferase Assay shows that PS1 FAD mutants significantly decrease the γ‐secretase‐mediated epsilon cleavage of VEGFR2 when compared to WT‐PS1, leading to the production of significantly lower amount of the VEGFR2/CTF2 peptide indicating a loss of γ‐secretase proteolytic function. This loss of function is comparable to the effect of γ‐secretase inhibitor RO4929097. Co‐IP experiments show that PS1 forms complexes with VEGFR2 and BiFc experiments show that PS1 binds to VEGFR2. Docking analysis indicates that the binding is electrostatically favored. Co‐IP between the PS1‐FAD mutants and VEGFR2 show that FAD mutants do not have differential binding to the VEGFR2 compared to WT‐PS1, showing that the effect of these mutants on VEGFR2 cleavage is not due to altered binding. PS1 downregulation of endothelial cells indicates that PS1 plays a role in the regulation of the VEGFA‐induced phosphorylation of VEGFR2 at Y1175 and Y1054‐59 as well as p‐44/42 MAPK (Erk1/2) and in VEGFA‐induced tube formation *in vitro*.

**Conclusion:**

PS1 FAD mutants decrease production of signaling peptide VEGFR2/CTF2 probably due to reduced γ‐secretase activity of FAD mutants. Interestingly FAD mutations do not seem to affect the PS1‐VEGFR2 association. Our data support that PS1 regulates downstream signaling and angiogenic functions of VEGFR2.

